# Removal of Benzene and Toluene from Synthetic Wastewater by Adsorption onto Magnetic Zeolitic Imidazole Framework Nanocomposites

**DOI:** 10.3390/nano12173049

**Published:** 2022-09-02

**Authors:** George Z. Kyzas, Gordon McKay, Tariq J. Al-Musawi, Sabereh Salehi, Davoud Balarak

**Affiliations:** 1 Department of Chemistry, International Hellenic University, GR-654 04 Kavala, Greece; 2 Division of Sustainable Development, College of Science, Engineering and Technology, Hamad Bin Khalifa University, Qatar Foundation, Doha P.O. Box 5825, Qatar; 3 Al–Mustqbal University College, Building and Construction Engineering Technologies, Babylon 51001, Iraq; 4 Student Research Committee, Zahedan University of Medical Sciences, Zahedan 9816743463, Iran; 5 Department of Environmental Health Engineering, Health Promotion Research Center, Zahedan University of Medical Sciences, Zahedan 9816743463, Iran

**Keywords:** zeolite imidazolate framework, Fe_3_O_4_, benzene, toluene, adsorption, desorption

## Abstract

Considering the risk associated with exposure to benzene and toluene in water resources, researchers have been motivated to conduct studies to remove them from aqueous solutions. Thus, by performing the present study, the potential of Fe_3_O_4_/zeolite imidazolate framework nanoparticles (Fe_3_O_4_@ZIF-8) was evaluated for the adsorption of benzene and toluene. Accordingly, the solution pH, Fe_3_O_4_@ZIF-8 dosage, mixing time, concentration of benzene and toluene, and temperature, were the parameters considered for conducting the batch experiments, for which their effect on adsorption efficiency was evaluated. Our conducted experiments introduced the neutral pH as the best pH range to obtain the maximum removal. Fitting the adsorption data into the various models revealed the aptness of the Langmuir isotherm equation in describing experimental information and highest adsorption capacity; for benzene it was 129.4, 134.2, 137.3, and 148.2 mg g^−1^, but for toluene it was 118.4, 125.2, 129.6, and 133.1 mg g^−1^, for temperature 20, 30, 40, and 50 °C, respectively. Using obtained optimal conditions, the adsorption efficiencies of benzene and toluene were obtained to be 98.4% and 93.1%, respectively. Kinetic studies showed acceptable coefficients for PSO kinetics and confirmed its suitability. Also, the recyclability results showed that for six consecutive periods of the adsorption-desorption process, the percentage of removal decreased by only 6% for benzene and toluene. Moreover, calculating thermodynamic parameter changes for benzene and toluene removal confirmed the favorability and spontaneity of the studied process and its endothermic nature. Considering the above findings, Fe_3_O_4_@ZIF-8 was found to be an operative adsorbent for removing pollutants.

## 1. Introduction

The increase in excessive consumption of different sources present in the environment, due to technological advances in the industry, has led to an increase in its destruction [1,2]. One of these sources is oil and its extraction, which, due to its toxic compounds, threatens human and animal health and ruins the environment. The rapid development of this industry, the increase in the speed of oil extraction, and the emergence of related industries have worsened the pollution situation [3,4]. One of the oil product hydrocarbons contains compounds such as benzene and toluene, which are mono-aromatic groups and contaminate groundwater resources [5]. These compounds have an extensive application in industries as solvents for organic compounds and equipment cleaning [6]. Some of the ways they infiltrate groundwater include leakage of petroleum products from storage tanks, pipes, and improper landfills. Benzene has been introduced as the priority pollutant and definitive carcinogen (Group A), and toluene has been a carcinogen in group E [5,7]. Benzene has been introduced to be a genotoxic carcinogen (such as lung cancer) and has been associated with adverse hematological effects (leukemia or non-Hodgkin’s lymphoma) [8,9]. Moreover, neurological disorders, in addition to carcinogenic effects, have been reported for exposure to toluene [10]. Hence, it has been found that much attention should be given to the control and elimination of these compounds in groundwater, and efforts should be taken to introduce effective techniques to remove them.

To remove these types of pollutant, various techniques have been subjected to study [11,12,13,14,15]. According to the results of the studies that employed the above-mentioned techniques, adsorption is a method with high efficiency and high popularity [16]; low-cost operation, high uptake capacity, and absence of chemical sludge are the features which provided this popularity for the mentioned method [17,18]. Despite this, some drawbacks have been found for the application of the conventional adsorbent, i.e., activated carbon, which is indicative of the necessity for more research and attempts to introduce superior adsorbents [19]. Attempting to find low-cost adsorbents have led to employing available and low-cost substances such as agricultural plants, mineral wastes, etc. [20,21]. However, the adsorbents produced by these substances could not actually provide successful adsorbents because they did not have sufficient adsorption capacities and satisfactory removal efficiency [22,23].

Jiagwe et al. [24] reviewed some important granular activated carbon derived from biomass waste materials for water treatment. Production steps such as granulation with and without binders, as well as different carbonization and activation methods used to enhance strength and increase attrition resistance of the waste-based GAC, have been presented. The use of these GACs for water treatment has exhibited a great potential, sometimes performing better than commercial carbons, depending on the target contaminant. Also, Chen et al. [25] reviewed the conversion of crayfish-shell derivatives to functional materials and their environmental applications. Crayfish shells and their derivatives provide a cost-effective and sustainable platform for the functional utilization of biological materials. The primary components, such as chitin, protein, and N-acetyl-d-glucosamine, can be separated by modified chemical or biological methods. Furthermore, crayfish shells can be converted into functional carbon-based materials, which are extensively used as cheap and abundant adsorbents or mesoporous carbon supports in chemical reactions. However, based on the limited research on crayfish shells and their derived functional materials, more research attention is needed to solve the scientific and technical challenges of crayfish-shell utilization.

By continuation of the studies and research on this subject, nanomaterials such as carbon nanomaterials have been addressed to be associated with a high ability for removing the pollutant due to their excellent properties. For instance, Bina et al. (2012) used carbon nanotubes to remove benzene and toluene and reported favorable results [10]. Mohammadi et al. (2017) employed cupric oxide nanoparticles for removing benzene and toluene and found the removal efficiency of 98.7% for benzene and 92.5% for toluene, using their studied adsorbent [23]. Mahmud et al. (2018) studied the removal efficiencies of BTEX using iron nanoparticles and obtained satisfactory results for its application [26]. Despite the benefits, the toxicity and high production costs detected for these materials have led to restricting their application [27]. MOF is a type of nanomaterial which has recently found more applicability in different areas of science; this favorability of use is due to having exceptional features, e.g., large surface area, stability in water and high porosity [27,28]. ZIF-8 is a MOF with good stability in water. It is a microporous MOF with a framework structure, which has led to its being introduced as a suitable adsorbent for removing various types of pollutants [29,30]. Despite the advantages mentioned above, the problem associated with its separation (this problem rises due to the small size of ZIF-8 particles), which needs a long time and is difficult to achieve, limits its application [31,32]. Magnetic nanoparticles, i.e., Fe_3_O_4_, have a facile separability from solutions, which leads to increasing their popularity, since it leads to eliminating the processes such as centrifugation and filtration for removing these types of adsorbents from solutions. Its combination with ZIF-8 can lead to integrating the properties of these two nanoparticles so that the new composite is easily separated after adsorption [33]. Based on this, the Fe_3_O_4_/ZIF-8 has been studied as an adsorbent by many researchers. For example, Hue et al. (2018) used Fe_3_O_4_/ZIF-8 for the adsorption of As(III) from an aqueous solution. El-Desouky (2021) synthesized Fe_3_O_4_/ZIF-8 for the adsorption of anionic dyes [34]. Also, Qu et al. (2022) utilized this composite in another study as an adsorbent for removing phenol [35]. In all the studies, as mentioned earlier, Fe_3_O_4_/ZIF-8 was found to be an effective and excellent adsorbent with remarkable surface area, high reusability, and recyclability. Furthermore, Ma et al. [36] investigated the ZIF-67@wood obtained by in situ growth of ZIF-67 on wood, which is carbonized to prepare magnetic WC-Co composites. The hierarchical porous structure of wood can facilitate the rapid passage of dye solution, and promote full contact between magnetic core–shell Co/C nanoparticles and dyes. The adsorption capacities of CR and MB by Co/C-1000 are 1117.03 and 805.08 mg g^−1^, respectively.

Based on the above, since benzene and toluene are toxic and harmful compounds for different sectors of the life cycle, and there is no report for employment of Fe_3_O_4_/ZIF-8 in the adsorption process for removing the mentioned pollutant, the present study assessed the ability of Fe_3_O_4_/ZIF-8 in adsorption of benzene and toluene. Moreover, characteristics of the prepared adsorbent were identified using some common analyses. In addition, studies related to the effect of parameters (pH, Fe_3_O_4_@ZIF-8 dosage, mixing time, concentration of pollutant, and temperature), isotherm, kinetic, and thermodynamic of the adsorption process, which is imperative and necessary in the adsorption process, were also conducted. Finally, evaluating the reusability of the Fe_3_O_4_/ZIF-8 was another object of the present study.

## 2. Materials and Methods

### 2.1. Materials

Benzene and toluene (analytical grade and purity of greater than 99%) were employed as target pollutants in this study; these chemicals were provided by Merck and were used with no further purification. Other chemicals required for conducting the present study were hexahydrate zinc nitrate (Zn(NO_3_)_2_∙6H_2_O, 98%), 2-methylimidazole (C_4_H_6_N_2_ 99%), ammonium hydroxide (NH_4_OH), sodium hydroxide (NaOH, ≥98%), methanol (CH_3_OH), ≥99%), hydrochloric acid (HCl, ≥36.5%), ammonium ferrous sulfate ((NH_4_)_2_(FeSO_4_)_2_·6H_2_O and ferric chloride, which was supplied by Sigma Aldrich (St. Louis, MO, USA).

### 2.2. Synthesis of Fe_3_O_4_

Synthesizing Fe_3_O_4_ nanoparticles was done based on a modified co-precipitation process using Fe^3+^ and Fe^2+^ [22]. First, a solution with a 1:2 molar ratio between Fe^2+^ and Fe^3+^ should be formed; this was done by liquefying ferrous ammonium sulfate ((NH_4_)_2_(FeSO_4_)_2_·6H_2_O, 1.97 g, 5 mmol) and ferric chloride (FeCl_3_·6H_2_O, 1.622 g, 10 mmol) in distilled water (100 mL). Instilling ammonia solution under a nitrogen blanket (NH_3_·H_2_O, 28 percent, 0.7 M) was done after 20 min. Mechanical stirring was applied for the formation of the precipitate, and heating was then carried out for 30 min at 80 °C. After centrifuging nanoparticles and cooling them to room temperature, they were washed three times with distilled water.

### 2.3. Synthesis of Zeolitic Imidazole Framework-8 (ZIF-8)

Producing ZIF-8 in the present study was achieved according to results reported in a previous study [31]; based on this, Zn(NO_3_)_2_∙6H_2_O (2.97 g) was first poured into double distilled water (3 mL). Then, 2-methylimidazole (1.64 g) was added to the NH_4_OH solution (20.75 mL). Finally, two prepared solutions, i.e., Zn(NO_3_)_2_ and 2-methylimidazole, were mixed. The combination of mentioned solutions led to the immediate formation of milky suspension, which was agitated for 10 min at room temperature and led to its crystallization. After completion of the process, collecting the final product was done by centrifugation. It was then rinsed with deionized water (three to four times) until reaching a pH of around 7. Finally, the drying process at 60 °C was applied for the resultant product.

### 2.4. Synthesis of Fe_3_O_4_@ZIF-8

**Figure 1:**
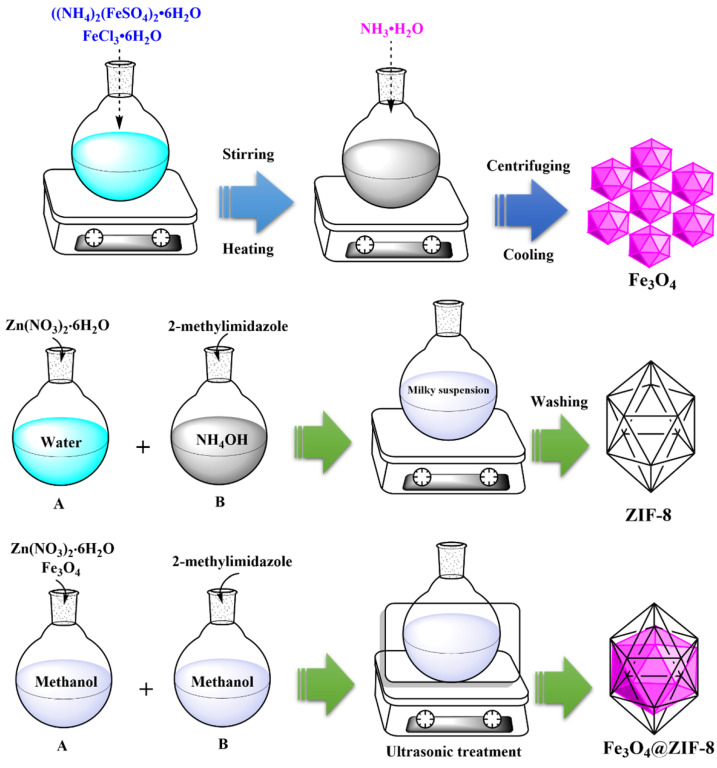
Synthesis route of Fe_3_O_4_, ZIF-8 and Fe_3_O_4_@ZIF-8.

2 mmol, 0.595 g of Zn(NO_3_)_2_∙6H_2_O, and 0.05 g Fe_3_O_4_ were dissolved as (solution A) in 7.5 mL of methanol. Then, solution B was prepared by liquefying 2-methylimidazole (0.65 g, 8 mmol) in 7.5 mL of methanol. After preparing the above solutions, solution A was instilled into solution B under ultrasonic treatment. The resultant obtained from the reaction between those solutions was brown Fe_3_O_4_@ZIF-8 precipitate, which was collected by centrifugation process. It was then dried at 60 °C. The whole synthesis route is illustrated in Figure 1.

### 2.5. Adsorption Experimental Design

To conduct the adsorption experiments of the studied pollutants, a determined amount of Fe_3_O_4_@ZIF-8 was poured into glass flasks, which were filled with 100 mL of the pollutants being investigated. After covering the glass flasks with Teflon caps, they were exposed to stirring in a thermostatic shake water bath, at 150 rpm for a certain amount of time. For regulating the pH of the studied solution, NaOH (0.1 M) and HCl (0.1 M) were employed. After completion of the process, an external magnet was utilized to separate Fe_3_O_4_@ZIF-8. Calculating the adsorption capacity (q_e_, mg g^−1^) and the removal percentage (%) was achieved using the below equations [37]:

(1) $$\mathrm{Qe}\;=\frac{\left(C_{0}-C_{e}\right)\times V}{m}$$

(2) $$R\;\left(\%\right)=\left(\frac{C_{0}-C_{e}}{C_{0}}\right)\times 100\%$$

Determining the initial and equilibrium concentrations of the studied pollutants in solutions was carried out utilizing Shimadzu visible spectrophotometer (DR5000); this test was conducted at wavelengths of 206 and 254 nm for toluene and benzene, respectively.

### 2.6. Devices Used in the Study

The following devices were used for adsorbent analysis: TEM model (LEO 912 AB) and SEM model (Mira 3-XMU), Fourier transform Infrared (FTIR) spectroscopy (Thermo Nicollet AVATAR5700), surface-area analyzer (ASAP2020, USA), vibrating sample magnetometer (Micromeritics Instrument Corp., Norcross, GA, USA), and X-ray diffraction (XRD, Rigaku D/Max 2500, Tokyo, Japan).

## 3. Results and Discussion

### 3.1. Characterizations

**Figure 2:**
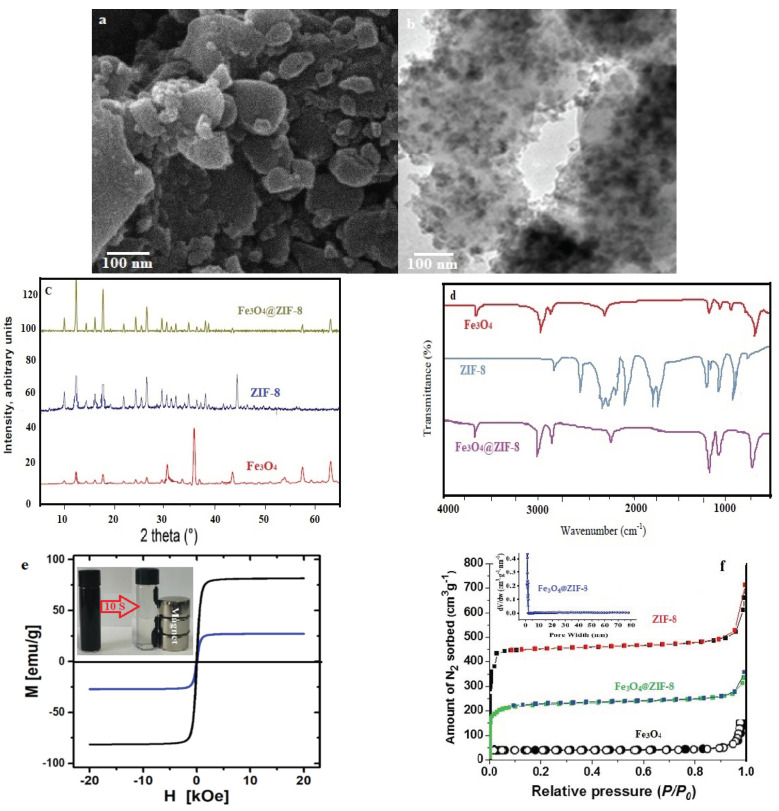
SEM patterns (**a**) TEM patterns, (**b**) XRD patterns, (**c**) FTIR spectra, (**d**) Magnetization curves, (**e**) Adsorption-desorption isotherms and (**f**) BJH pore size distribution of Fe_3_O_4_@ZIF-8.

According to Figure 2a (the SEM image of the Fe_3_O_4_@ZIF-8 composite), an uneven surface was observed for the Fe_3_O_4_@ZIF-8 composite. Moreover, according to TEM images (Figure 2b), a distribution of magnetic nanoparticles on the crystal surfaces of ZIF-8 was approved.

Diffraction of X-ray (XRD) was another test employed in this study to additionally validate the loading of Fe_3_O_4_ on ZIF-8 (Figure 2c). There were similarities between the XRD of Fe_3_O_4_@ZIF-8 and ZIF-8; this indicates the constancy of the ZIF-8 crystals’ sodality structure, even after synthesizing the Fe_3_O_4_ particles. Except for three peaks observed at 2θ = 33.4, 35.7, and 43.6° corresponding to the (220), (311), and (400) magnetite crystal facets (JCPDS No. 19-0629), which implied the high purity of the final Fe_3_O_4_@ZIF-8 particles, there were no other peaks [31].

FT-IR (in the range of 400.0 to 4000.0 cm^−1^) was another test used in the present study; this test evaluates the functional groups of the studied adsorbent. In Figure 2d, FT-IR spectra for ZIF-8, Fe_3_O_4_, and Fe_3_O_4_@ZIF-8 were provided. The peak was observed at 2922 cm^−1^ is an indicative of C-H group and OH stretching in carboxylic group (usually overlaps C-H). However, no carbonyl group was observed in the range 1710–1740 cm^−1^. In the distributed deep peak, the Fe-O bond absorption is featured at 614 cm^−1^. FT-IR spectrum of ZIF-8 represented the peaks of 2500–3500 cm^−1^ (the vibrations of the ZIF-8 pairs), 1384 cm^−1^ ((C-N) vibration), and peaks of 1667 cm^−1^ and 1580 cm^−1^ (the bending and stretching N-H vibration in the imidazole). However, the bands at 1350–1500 cm^−1^ were correspondent with the complete stretching ring. In addition, the peak of 421 cm^−1^ is raised by the Zn-N stretch mode [27]. Considering the results, it is clear that except for the peak at 614 cm^−1^, which is in correspondence with the Fe–O bonds, there is a closeness between the FT-IR spectrum of Fe_3_O_4_@ZIF-8 and ZIF-8. In conclusion, the successful production of composites is inferred by the above results.

According to the magnetic hysteresis loops measured for Fe_3_O_4_ and Fe_3_O_4_@ZIF-8 (shown in Figure 2e), there is a superparamagnetic characteristic for the composite with insignificant remanent magnetization at zero external magnetic field. Fe_3_O_4_ exhibited a saturation magnetization of 81.7 emu g^−1^, which diminished to 26.9 emu g^−1^ after loading ZIF-8; this diminution is described based on the addition of ZIF-8, which is a non-magnetic substance. The robust magnetic response facilitates the quick separation (<10 s) of Fe_3_O_4_@ZIF-8 particles from an aqueous solution using a magnet. Moreover, through mild agitation in the absence of a magnetic field, it is possible to redisperse the composite particles after adsorptive removal.

Figure 2f represents the results of nitrogen sorption isotherms, which were achieved for ZIF-8, Fe_3_O_4_, and Fe_3_O_4_@ZIF-8 at 77 K. As anticipated, a type I isotherm characteristic of microporous solids was detected for pure ZIF-8 (23). Considering the results of the BET model, a specific surface area of 1394 m^2^ g^−1^ was obtained for ZIF-8. Furthermore, for Fe_3_O_4_, the average pore diameter, pore volume, and specific surface area, were observed to be 5.41 nm, 0.077 cm^3^ g^−1^, and 21.2 m^2^ g^−1^, respectively. Nevertheless, the values of the above parameters for Fe_3_O_4_@ZIF-8 were detected to be 3.17 nm, 0.612 cm^3^ g^−1^, and 942 m^2^ g^−1^, respectively. After loading ZIF-8 on the surface of Fe_3_O_4_, results were representative of an enhancement in the pore volume and specific surface area of Fe_3_O_4_@ZIF-8 and a decline in pore size, which leads to enhancing active sites on Fe_3_O_4_@ZIF-8 and developing the adsorption of target pollutants from water. In addition, 67.5 wt.% shares of Fe_3_O_4_@ZIF-8 composite were related to the ZIF-8.

### 3.2. Influence of Parameters

One of the critical factors introduced by different studies for applying adsorbents in the adsorption process, is solution pH. The proton transfer may happen on the surface of adsorbents under different pH values, which results in adsorption in reaction pathways [37]. Determination of the optimum solution pH was done based on changing its values from 3 to 11 using an initial concentration of 100 mg∙L^−1^ and a Fe_3_O_4_@ZIF-8 concentration of 0.75 g L^−1^, and selecting the pH with the best adsorption efficiency. According to the results of this part, represented in Figure 2a, the removal efficiencies at pH of 3 and 7 were about 75.7% and 98.4% for benzene and 64.3% and 93.1% for toluene, respectively, which suggests the increasing trend for adsorption efficiency until pH of 7. However, a continuous increase in pH was observed to be associated with a diminution of adsorption efficiency. Based on the above observations, a pH of 7 was the optimum pH value for benzene and toluene adsorption and was considered optimal for the next experiments.

Adsorption decreases at high pH, which could be attributed to the competition between benzene and toluene and hydroxide (OH^−^) ions for same active sites available on the surface of Fe_3_O_4_@ZIF-8. Additionally, the lower adsorption of Fe_3_O_4_@ZIF-8 at extremely acidic (3–5) and basic (9–11) conditions is attributed to the surface polarity and hydrogen bonding between adsorbent and adsorbate [23]. Moreover, the surface groups (–COOH) are of paramount importance for binding of hydrophobic contaminants and the solution pH affected the surface charges through protonation and deprotonation of the organic compounds. At neutral conditions, strong electrostatic interactions occur due to the interactions of oxygen-bearing functional groups (with a negative charge) on Fe_3_O_4_@ZIF-8 and π electrons of ring structures (with a positive charge), which develops the adsorption efficiency; based on this, the electrostatic interaction has a role in the adsorption process [37].

**Figure 3:**
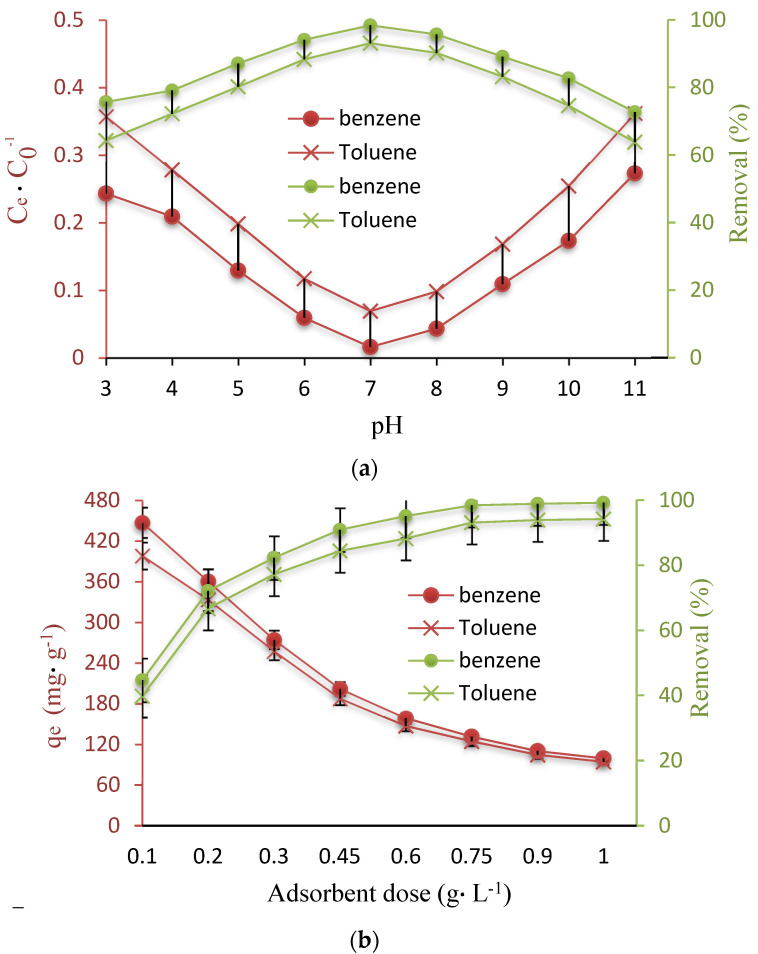

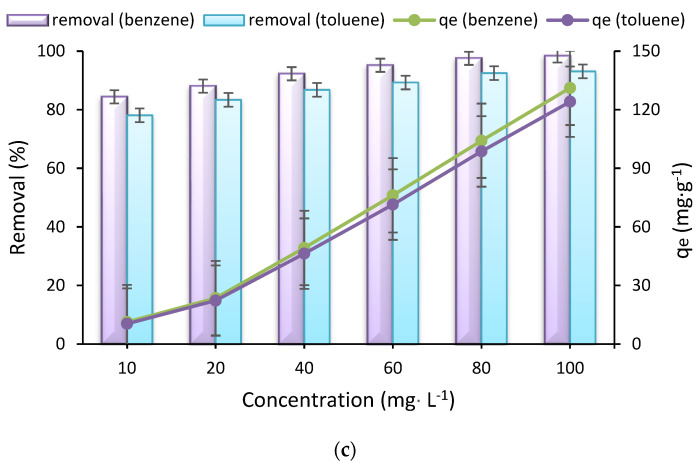
(**a**) Effect of pH on benzene and toluene removal, (**b**) Effect of Fe_3_O_4_@ZIF-8 dose, (**c**) Effect of concentration on capacity adsorption and removal.

The adsorbent dose has been found to be an important factor since it is able to represent the potential of the adsorbent, i.e., Fe_3_O_4_@ZIF-8, for a known initial benzene and toluene concentration. Therefore, the studies to determine the changes in benzene and toluene adsorption, by changing the Fe_3_O_4_@ZIF-8 dosage, were conducted using 0.1 to 1 g L^−1^ of the adsorbent at a pH of 7, and initial benzene and toluene concentration of 100 mg L^−1^ for 75 min. According to the results of this part (Figure 3b), there is an association between benzene and toluene removal efficiency and the studied factor, i.e., Fe_3_O_4_@ZIF-8 dosage; based on this, an increase in the adsorbent dosage from 0.1 to 0.75 g led to an improving removal efficiency from 44.7% to 98.4% for benzene and from 39.8% to 93.1% for toluene. In addition, a rapid enhancement was detected in benzene and toluene removal capacity by increasing the Fe_3_O_4_@ZIF-8 dose from 0.1 to 0.75 g. To explain the obtained result, it should be mentioned that although the number of available sites develops by increasing the adsorbent dosage, they remain unsaturated in the adsorption process [38]. Moreover, dosages above 0.75 g L^−1^ exhibited a slight effect on the adsorption capacity of Fe_3_O_4_@ZIF-8; according to this, 0.75 g L^−1^ was considered as the optimum dosage.

The results related to the effect of initial benzene and toluene concentration, represented in Figure 2c, depict that there is a link between rising the initial pollutant concentration and the removal efficiency and adsorption capacity. For instance, removal efficiencies for initial benzene concentrations of 10 mg L^−1^ and 100 mg L^−1^ (contact time 75 min, pH = 7, dose 0.75 g L^−1^, and temperature 30 °C) were more than 84.4% (with an adsorption capacity of 11.25 mg g^−1^) and 98.4% (with an adsorption capacity of 131.2 mg g^−1^), respectively. This trend was also perceived for toluene. Above mentioned observations confirm the dependence of the removal efficiency of studied pollutants by Fe_3_O_4_@ZIF-8 on their concentrations. Enhancing the driving force related to the concentration gradient has been reported as the reason for developing adsorption capacity, due to increasing the initial benzene and toluene concentration at a fixed dose of adsorbent [39,40]. Based on this, initial concentrations of the pollutant molecules are considered as effective factors to enhance driving force, which can participate in overcoming the mass transfer resistance of all molecules between the aqueous and solid phases.

### 3.3. Investigation of Contact Time and Kinetic Studies

**Figure 4:**
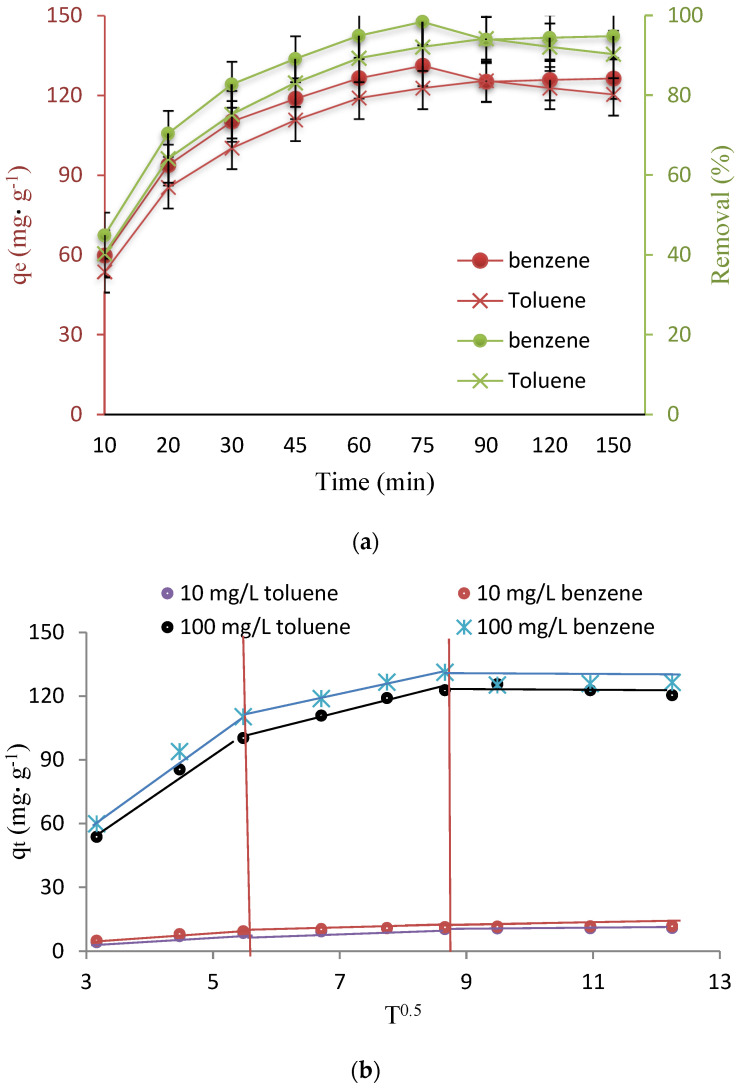
(**a**) Effect of contact time on capacity adsorption and (**b**) IPD plots.

Equilibration time in adsorption studies is one of the most important parameters, which should be determined. The high adsorption rate in the early stages of adsorption can be due to the high driving force and fast transfer of toluene and benzene molecules, and the adsorbent surface. After 75 min for benzene and 90 min for toluene, the amount of absorption decreases to some extent (Figure 4a). The reason for this can be the separation of some toluene and benzene from the adsorbent during the desorption stage, and the reduction of available active sites. This state is caused by the lower dissolution rate of toluene (530 mg L^−1^), compared to benzene (1970 mg L^−1^), the higher molecular weight of toluene (92 g mol^−1^) than benzene (78 g mol^−1^), and the higher boiling point of toluene (110.7), compared to the boiling point of benzene (80.1) [3].

Also, the evaluation of the kinetic parameters of the process was carried out through the PFO and PSO, IPD, and Elovich kinetic equations [39,40,41]. The equations related to the isotherms were shown in Table 1. Examining the parameter values of PFO and PSO equations of toluene and benzene adsorption process by Fe_3_O_4_@ZIF-8, in Table 1, showed that, based on the correlation coefficient values, the data obtained from the adsorption test are more consistent with the PSO model compared to the PFO. Also, the results showed that the correlation coefficient of PSO is very high and the value of q_e_ cal is close to the value of q_e_ exp. Therefore, the adsorption process follows the PSO model, which indicates the chemical interaction between the adsorbent and the adsorbate.

Determining the mechanism and the controlling or limiting step of the ion exchange process is usually done through the IPD model. The rate constants of the adsorption of toluene and benzene ions by studied Fe_3_O_4_@ZIF-8, according to the mentioned model, were shown in Figure 4b, which represents three stages in the process of toluene and benzene ions adsorption by Fe_3_O_4_@ZIF-8: it includes the first stage, i.e., the transfer of toluene and benzene ions from the solution to the liquid film around the adsorbent; the second step, i.e., the mentioned ions reach the adsorbent surface from the film; and the third step, i.e., their penetration from the surface to the internal sites of Fe_3_O_4_@ZIF-8 [42,43]. By comparing the trend of the slope of each stage, it can be seen that the first stage, i.e., the arrival of toluene and benzene ions to the film around the adsorbent, is carried out with the highest rate for which this phenomenon is possible, mostly by stirring the solution.

### 3.4. Isotherm Studies

In this research, the mechanism and behavior of toluene and benzene ions’ adsorption on Fe_3_O_4_@ZIF-8 were studied by examining the equilibrium and kinetics of the process. To determine and check the isotherm model governing the process, after analyzing the concentration of toluene and benzene in the samples, the equilibrium data were fitted on the linear form of D-R-, Temkin, Freundlich, and Langmuir models, and after deriving the equation of the fitting line, the constants related to each was calculated and determined [44,45,46]. The equations and parameters calculated related to the isotherms were shown in Table 2. According to this table, almost all the models examined have a good fit on the laboratory data, so that the correlation coefficient is above 0.9 in all cases. Also, the R^2^ value for the Langmuir models was calculated to be higher than 0.99, which indicates the fit on the data and the use of their constants to describe the process.

The extracted data of the Langmuir model show that the maximum adsorption capacity for benzene was 129.4, 134.2, 137.3, and 148.2 mg g^−1^, and for toluene was 118.4, 125.2, 129.6, and 133.1 mg g^−1^, for temperature 20, 30, 40, and 50 °C, respectively. On the other hand, the proper fit of the data on the Freundlich model indicates that the process is not limited to a specific surface of the adsorbent, and that the surface for studied benzene and toluene has heterogeneity with different adsorption energies. Researchers have always tried to link the constant parameters in the model (1/*n* and K_F_) with the adsorption mechanism. In general, in the Freundlich model, 1/*n* and K_F_ are constants that describe all the factors affecting the adsorption capacity and the desirability of the adsorption process on the adsorbent, respectively. When the value of *n* is in the range of 1 to 10, the adsorption process is classified in the desired class [47,48]. The higher value of n (the lower value of 1/*n*) represents the more significant inhomogeneity of the absorbent surface for the pollutant. According to the data in the above table, the numerical value of 1/*n* for the investigated adsorbent for benzene and toluene is lower than one, which indicates that the process is favorable and adsorption of benzene and toluene ions from the solution is easy [49]. The comparison of the adsorption capacity obtained from this study with similar studies was shown in Table 3, and as can be seen, it has a good adsorption capacity compared to the adsorbent used in this study.

The DR model is another isotherm equation that the laboratory data obtained followed this model with a high correlation coefficient. This model is generally used to describe the adsorption mechanism [50,51]. This model’s constant value of E may be used to determine whether the process is physical or chemical. This value represents the average amount of energy required to remove the molecules of the adsorbed species from the surface of the adsorbent. When the value of E is less than 8 kJ mol^−1^, the process is a physical process, and when it is in the range of 8 to 16 kJ/mol, it is a chemical process [52,53,54]. As can be seen in the data table of the used models, this value for benzene and toluene adsorption is higher than 8 kJ mol^−1^, which indicates the dominant role of chemical adsorption in the adsorption process of benzene and toluene on Fe_3_O_4_@ZIF-8 adsorbent.

### 3.5. Effect of Temperature and Thermodynamics Study

**Figure 5:**
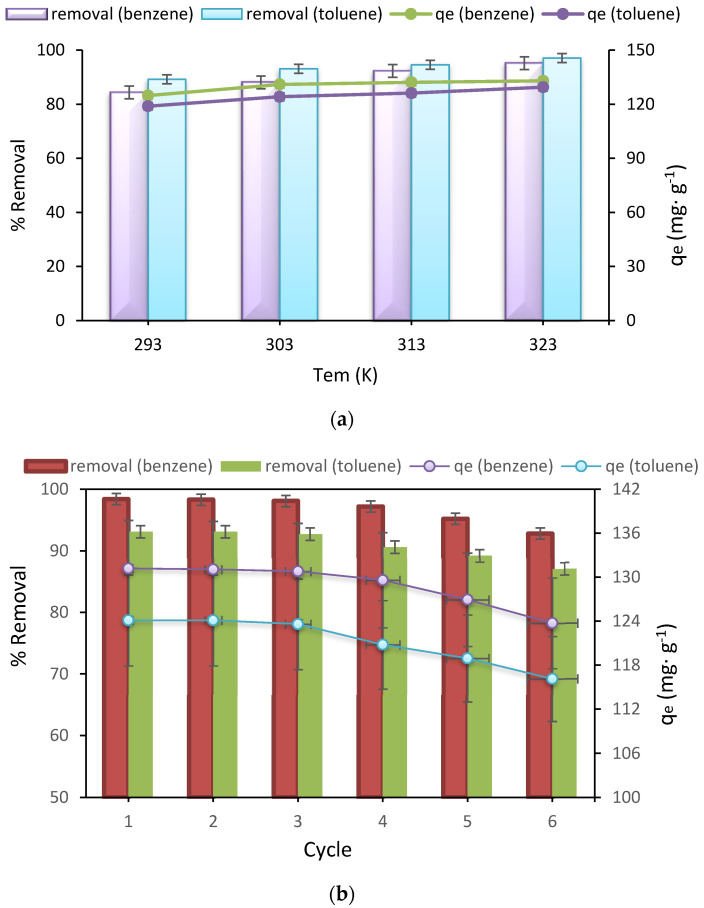
(**a**) Effect of temperature, (**b**) Reusability study of Fe_3_O_4_@ZIF-8 on benzene and toluene uptake.

The results of the effect of temperature are shown in Figure 5a, and as it is quite clear, with the increase in temperature from 20 to 50 °C, the removal percentage and adsorption capacity for both benzene and toluene increases, which shows that it is endothermic adsorption of benzene and toluene [55,56].

Thermodynamic studies were conducted based on related parameters, i.e., ΔG^0^, ΔH^0^, and ΔS^0^ at different temperatures. The adsorption process is represented by the following [57,58,59].

ΔG^0^ = −RT ln K_c_

$$\ln\left(K_{c}\right)=\frac{{\Delta S}^{0}}{R}-\frac{{\Delta H}^{0}}{\mathrm{RT}}$$

$$K_{c}=\frac{q_{e}}{C_{e}}$$

Considering the slopes and intercepts obtained from plotting a graph of ln Kc vs. 1. T^−1^, estimating ΔH^0^ and ΔS^0^ is performed. According to the results, the adsorption has endothermic nature considering the positive values obtained for ΔH° value (ΔH° = 35.07 kJ mol^−1^ for benzene and ΔH^0^ = 79.21 kJ mol^−1^ for toluene) [60,61,62,63]. However, the positive values of ΔS^0^ (ΔS^0^ = 0.139 kJ mol^−1^K^−1^ for benzene and ΔS^0^ = 0.295 kJ mol^−1^K^−1^ for toluene) are representative of the increasing entropy of the system during the adsorption process [64,65]. It shows that our studied process is a somewhat temperature-dependent process. Moreover, at the studied temperature (at 293, 303, 313, and 323 K), ΔG^0^ values for benzene adsorption were −5.82, −7.25, −8.19, and −10.17 kJ mol^−1^, while the values for toluene adsorption were −7.23, −10.78, −12.95, and −16.35 kJ mol^−1^, respectively. The negative values for ΔG° were indicative of the spontaneous nature of the evaluated adsorption, and increasing temperature enhances the adsorption capability of benzene and toluene [66,67,68].

### 3.6. Adsorbent Recyclability

In this study, adsorption-desorption tests were performed in six different stages to recover the adsorbent under study. Thus, after adsorption, the Fe_3_O_4_@ZIF-8 was separated using a magnet, and the separated adsorbent was washed with distilled water and methanol in a ratio of one to one. Then, it was placed in the ultrasonic device for 30 min, and the magnetic separation was again performed using a magnet. After desorption, the adsorption phase began. In this section, the optimal parameters obtained in the results section were used. The results were shown in Figure 5b, and as can be seen, after six successive adsorption-desorption runs, the removal percentage for benzene changed from 98.4 to 92.8%, and the adsorption capacity of 123.7 mg g^−1^ in the initial adsorption reached 116.1 mg g^−1^. Also, the removal percentage of toluene in six consecutive steps attained from 93.1 to 87.1%, and the adsorption capacity in the first step was 124.1 mg g^−1^, and for the sixth run, it was 116.1 mg g^−1^. Therefore, for the removal of toluene in six steps, a 6% reduction in removal was observed, and for benzene in six steps, a 5.6% reduction in removal was observed, which is a very acceptable value. This decrease in removal can be due to the loss of the adsorbent during washing and the decrease in the amount of adsorbent, and the lack of complete desorption of the adsorbent in the desorption stage, which reduces the active sites of the adsorbent and subsequently removal efficiency [69,70].

## 4. Conclusions

This study was to evaluate the efficiency of a magnetic nanocomposite (Fe_3_O_4_@ZIF-8) for adsorbing two pollutants, i.e., benzene and toluene; mentioned nanocomposite was prepared using Fe_3_O_4_ and ZIF-8, according to the co-precipitation method. According to the results, a high surface area (942 m^2^ g^−1^) was detected for Fe_3_O_4_@ZIF-8 composites. Isotherm studies were indicative of the suitability of the Langmuir model for expressing the adsorption isotherm; based on this, monolayer adsorption is detected for the uptake of benzene and toluene on Fe_3_O_4_@ZIF-8. The comparison of R^2^ values (0.99 for both pollutants) revealed the suitability of PSO for fitting the data obtained from studies related to adsorption kinetics. Considering the thermodynamic studies, the adsorption process is a spontaneous and endothermic. Considering the results of this research, due to the acceptable efficiency of Fe_3_O_4_@ZIF-8 in the adsorption of benzene and toluene, and its easy separation after the process, it has been found to be a nanocomposite for practical application.

## Figures and Tables

**Table 1:** Kinetic parameters for the adsorption of benzene and toluene on Fe_3_O_4_@ZIF-8.

Models	Benzene (mg L^−1^)		Toluene (mg L^−1^)		Models	Benzene (mg L^−1^)		Toluene (mg L^−1^)	
	10	100	10	100		10	100	10	100
PFO	$\mathrm{Log}\left(q_{e}-q_{t}\right)={\mathrm{Logq}}_{e}-\frac{k_{1}}{2.303}t$				Elovich	$q_{t}=\frac{1}{b}\ln\left(\mathrm{ab}\right)+\frac{1}{b}\ln\left(t\right)$			
q_e (calf)_ K_1_ R^2^	4.2 0.027 0.895	83.1 0.091 0.871	3.9 0.019 0.925	77.2 0.044 0.897	α × 10^6^ b R^2^	0.041 7.24 0.921	0.73 19.6 0.898	0.025 5.32 0.903	0.57 16.2 0.987
PSO	$\frac{t}{q_{t}}=\frac{1}{k_{2}q_{e}^{2}}+\frac{1}{q_{e}}t$				IPD	$q_{t}=k_{b}\times t^{0.5}+C$			
q_e (cal)_ K_2_ R^2^	13.8 0.002 0.998	126.4 0.003 0.996	11.2 0.001 0.994	114.4 0.002 0.992	K_b_ C R^2^	2.13 24.2 0.876	6.16 65.8 0.658	1.94 17.4 0.805	6.68 55.5 0.714

**Table 2:** Isotherm parameters for adsorption of benzene and toluene at various temperatures.

	Benzene				Toluene			
models	293 K	303 K	313 K	323 K	293 K	303 K	313 K	323 K
Q_e_ exp (mg g^−1^)	124.8	130.9	132.1	132.9	118.9	124.1	126.2	129.4
**Langmuir**	$\frac{1}{q_{e}}\;$ $=\frac{1}{q_{m}\;}\;$ $+\frac{1}{K_{L}C_{e}q_{m}}$				$R_{L}=\frac{1}{1+K_{L}C_{0}}$			
q_m_ (mg g^−1^) K_L_ (L mg^−1^) R_L_ R^2^	129.4 0.0024 0.806 0.989	134.2 0.0028 0.781 0.993	137.3 0.0036 0.735 0.996	148.2 0.0041 0.709 0.997	118.4 0.0018 0.847 0.989	125.2 0.0021 0.826 0.989	129.6 0.0023 0.813 0.989	133.1 0.0026 0.793 0.989
**Freundlich**	$\log\;q_{e}=\frac{1}{n}$ log Ce + log K_F_							
K_F_	7.02 0.389 0.862	6.51 0.354 0.891	7.23 0.342 0.873	7.89 0.425 0.823	10.1 0.342 0.834	12.3 0.453 0.786	9.82 0.511 0.821	8.41 0.562 0.792
1/n								
R^2^								
**Temkin**	$q_{e}=B\;\ln\;K_{T}+B\;\ln\;C_{e}$							
K_T_ (L g^−1^) Β (J mol^−1^) R^2^	2.16 12.1 0.841	1.76 17.4 0.834	1.73 24.8 0.865	1.22 38.3 0.871	1.84 8.22 0.812	1.32 13.4 0.832	1.05 19.1 0.846	0.924 19.9 0.802
**D-R**	$\log q_{e}=\ln{\;q}_{m}-\beta \varepsilon^{2}$				ε $=\mathrm{RT}\;\mathrm{Ln}\;\left[1+\frac{1}{C_{e}}\right]$			
q_m_ (mg g^−1^) E R^2^	82.2 7.64 0.956	89.2 8.02 0.987	92.4 8.49 0.971	98.3 8.95 0.985	72.4 7.14 0.947	78.8 7.66 0.948	84.1 8.91 0.931	89.4 9.27 0.912

**Table 3:** Comparison of adsorption capacity of different adsorbents.

Adsorbent	Pollutant	$Q_{m}\;(\mathrm{mg}\;g^{-1})$	Reference
Iron nanoparticles	benzene	41.2	[26]
Iron nanoparticles	toluene	27.6	[26]
CuO nanoparticles	benzene	36.9	[23]
CuO nanoparticles	toluene	40.2	[23]
Activated carbon	benzene	51.2	[7]
Activated carbon	toluene	58.5	[7]
MWCNT	benzene	56.9	[10]
MWCNT	toluene	63.2	[10]
periodic organosilica	benzene	65.1	[15]
periodic organosilica	toluene	97. 6	[15]
Fe_3_O_4_@ZIF-8 Fe_3_O_4_@ZIF-8	benzene toluene	148.2 133.1	This study This study

## Data Availability

The data presented in this study are available on request from the corresponding authors.

## Nomenclature

C_0_ = concentrations of benzene and toluene in mg L^−1^ at the start
C_e_ = concentrations of benzene and toluene in mg L^−1^ at equilibrium
V = benzene and toluene volume at mL
m = Fe_3_O_4_@ZIF-8 masses in mg
K_L_ = (L mg^−1^) is the Langmuir adsorption constant
K_F_ = Freundlich constant (L g^−1^)
n = heterogeneity factor (If the value of n < 1, the adsorption is a chemical process; if n > 1, then it is a physical process).
q_e_ = adsorption capacity at equilibrium
q_m_ = maximum adsorption capacity
R_L_ = dimensionless separation factor
The values of R_L_ show whether the biosorption process is irreversible (R_L_ = 0), linear (R_L_ = 1), favorable (0 < R_L_ < 1) or undesirable (R_L_ > 1)
ΔG° = Gibbs free energy (kJ mol^−1^)
ΔS° = entropy (kJ mol^−1^ K^−1^)
ΔH° = enthalpy (kJ mol^−1^)
K_1_ = equilibrium rate constant for pseudo-first order kinetics (PFO) (1.min^−1^)
K_2_ = equilibrium rate constant for pseudo-second order kinetics (PSO) (g mg^−1^ min^mg^)
K_b_ =Intra-Particle Diffusion (IPD) rate constant (mg g^−1^ min^−1/2^)
C= thickness of the boundary layer
β= D-R isotherm constant (mol^2^ kJ^−2^)
ε= Polanyi potential
E = The adsorption energy (kJ.mol^−1^)
K_T_ = constant of equilibrium binding (L.mg^−1^)
B = Temkin constant associated with the heat of adsorption (J/mol)
D-R = Dubinin-Radushkevich
XRD = X-ray Powder Diffraction
FTIR= Fourier Transform Infrared Spectrometer
SEM = Scanning electron microscope
TEM = Transmission Electron Microscope
VSM = Vibrating Sample Magnetometer
BET = Brunauer–Emmett–Teller
MOF = Metal-organic framework
